# The DNA repair protein SHPRH is a nucleosome-stimulated ATPase and a nucleosome-E3 ubiquitin ligase

**DOI:** 10.1186/s13072-019-0294-5

**Published:** 2019-08-21

**Authors:** Joanna Brühl, Jonathan Trautwein, Agnes Schäfer, Uwe Linne, Karim Bouazoune

**Affiliations:** 1 Institut für Molekularbiologie und Tumorforschung (IMT), Biomedizinisches Forschungszentrum, Philipps-Universität Marburg, Hans-Meerwein-Strasse 2, 35043 Marburg, Germany; 2 Fachbereich Chemie und Synmikro, Gerätezentrum Massenspektrometrie und Elementanalaytik, Philipps-Universität Marburg, Hans-Meerwein-Strasse 4, 35043 Marburg, Germany

**Keywords:** SHPRH, DNA repair, SNF2, Nucleosome, Ubiquitination/ubiquitylation

## Abstract

**Background:**

Maintenance of genome integrity during DNA replication is crucial to the perpetuation of all organisms. In eukaryotes, the bypass of DNA lesions by the replication machinery prevents prolonged stalling of the replication fork, which could otherwise lead to greater damages such as gross chromosomal rearrangements. Bypassing DNA lesions and subsequent repair are accomplished by the activation of DNA damage tolerance pathways such as the template switching (TS) pathway. In yeast, the RAD5 (Radiation-sensitive 5) protein plays a crucial role in initiating the TS pathway by catalyzing the polyubiquitination of PCNA (Proliferation Cell Nuclear Antigen). Likewise, one of the mammalian RAD5-homologs, SHPRH (SNF2, histone linker, PHD, RING, helicase) mediates PCNA polyubiquitination. To date, the study of SHPRH enzymatic functions has been limited to this modification. It is therefore unclear how SHPRH carries out its function in DNA repair. Moreover, how this protein regulates gene transcription at the enzymatic level is also unknown.

**Results:**

Given that SHPRH harbors domains found in chromatin remodeling proteins, we investigated its biochemical properties in the presence of nucleosomal substrates. We find that SHPRH binds equally well to double-stranded (ds) DNA and to nucleosome core particles, however, like ISWI and CHD-family remodelers, SHPRH shows a strong preference for nucleosomes presenting extranucleosomal DNA. Moreover, nucleosomes but not dsDNA strongly stimulate the ATPase activity of SHPRH. Intriguingly, unlike typically observed with SNF2-family enzymes, ATPase activity does not translate into conventional nucleosome remodeling, under standard assay conditions. To test whether SHPRH can act as a ubiquitin E3 ligase for nucleosomes, we performed a screen using 26 E2-conjugating enzymes. We uncover that SHPRH is a potent nucleosome E3-ubiquitin-ligase that can function with at least 7 different E2s. Mass spectrometry analyses of products generated in the presence of the UBE2D1-conjugating enzyme reveal that SHPRH can catalyze the formation of polyubiquitin linkages that are either branched or associated with the recruitment of DNA repair factors, as well as linkages involved in proteasomal degradation.

**Conclusions:**

We propose that, in addition to polyubiquitinating PCNA, SHPRH promotes DNA repair or transcriptional regulation in part through chromatin ubiquitination. Our study sets a biochemical framework for studying other RAD5- and RAD16-related protein functions through the ubiquitination of nucleosomes.

**Electronic supplementary material:**

The online version of this article (10.1186/s13072-019-0294-5) contains supplementary material, which is available to authorized users.

## Introduction

**Fig. 1:**
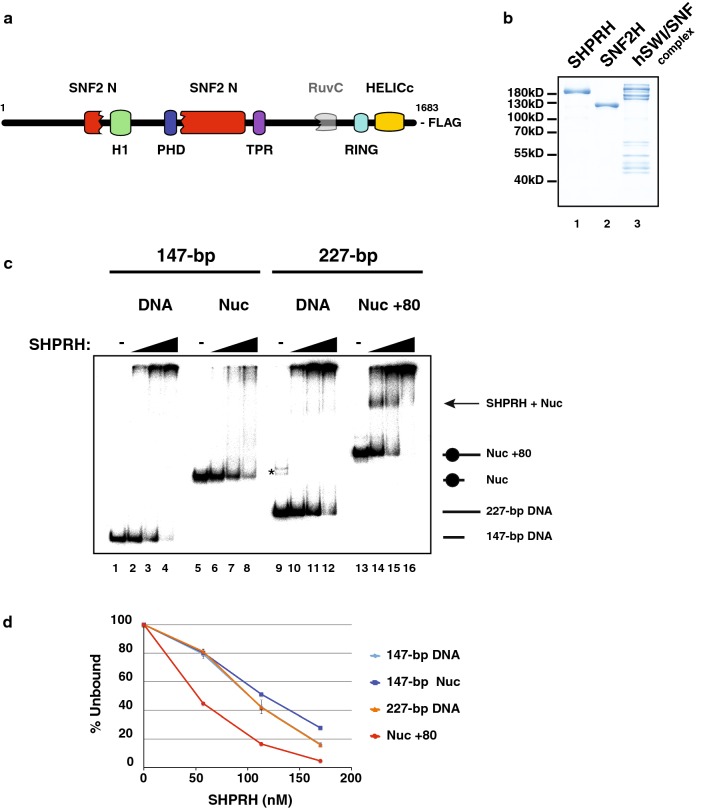
SHPRH is a dsDNA and a nucleosome-binding protein. **a** Schematic representation of the SHPRH protein domains (see main text for details). **b** SHPRH, SNF2H and the human SWI/SNF complex (~ 12 subunits) preparations which were used in assays were separated by SDS PAGE and stained with Coomassie blue. **c** Nucleosome electrophoretic mobility shift assay. 20 nM of radioactively labeled 147-bp DNA (lanes 1–4), the same DNA assembled into nucleosome core particles (lanes 5–8), **a** 227-bp DNA (lanes 9–12) or the same DNA assembled into mononucleosomes (Nuc + 80-bp of extranucleosomal DNA; lanes 13–16) were incubated with increasing concentrations of SHPRH (57, 113, 170 nM). Free or SHPRH-bound DNAs and nucleosomes were separated by native PAGE and visualized using a phosphorimager screen. The star indicates minor non-specific PCR-products. Positions of free DNA and nucleosomes (Nuc) are indicated on the right. **d** Quantification of the nucleosome electrophoretic mobility shift assay shown in (**c**). Percentages of the unbound DNA or nucleosome probes relative to the respective (−) SHPRH lanes (*Y*-axis) were plotted as a function of SHPRH concentration, as indicated on the *X*-axis. Error bars reflect standard deviation derived from two independent experiments. Note that error bars may not be visible for data with very small standard deviations

As its name indicates, the *SHPRH* gene encodes for a protein containing the following sequence features: a SNF2_N-terminal domain, a linker Histone (H1/H5)-like fold, a PHD zinc finger, a RING finger, and a helicase_C-terminal domain (Fig. 1a). This gene was initially identified as a candidate tumor suppressor, as it localizes to a chromosomal region (6q24) that is altered in several types of cancers ([1] and refs. therein). While other potential tumor suppressors have also been identified within this region (e.g., [2]), several studies have further linked SHPRH to cancer. For instance, axitinib, a tyrosine receptor kinase inhibitor used for the treatment of renal cell carcinoma, appears to act in part via SHPRH. A recent study suggests that axitinib stabilizes SHPRH which, in turn, increases the ubiquitination and degradation of β-catenin, a central coactivator of oncogenic Wingless-Type MMTV Integration Site (Wnt) responsive genes ([3] and for review [4]). Moreover, the *SHPRH* gene locus produces a circular RNA (circ-SHPRH) which is fully translated into a 146-amino acid polypeptide (SHPRH-146aa). This polypeptide acts as a decoy and therefore protects full-length SHPRH from degradation resulting from ubiquitination by the denticleless (DTL) E3 ligase. Overexpression of SHPRH-146aa in glioblastoma cells reduces their malignant behavior and tumorigenicity both in vitro and in vivo, further supporting the idea that SHPRH possess tumor suppressor functions [5]. In addition, recent analyses of bi-allelic alterations in The Cancer Genome Atlas dataset uncovered an association between microsatellite instability (MSI) and silencing of the SHPRH gene by DNA methylation. More specifically, silencing of *SHPRH* is associated with one of the characteristic cancer mutational signatures (mutational signature 6), which is common in uterine cancer. Interestingly, Signature 6 is also associated with mutations in DNA mismatch repair genes and is found in tumors showing elevated MSI [6].

A role for SHPRH in preventing genomic instability is consistent with tumor suppressor functions. While SHPRH has so far not been involved in DNA mismatch repair, it is now well-established that SHPRH contributes to the maintenance of genome integrity via a DNA damage tolerance (DDT) or post-replication repair (PRR) pathway. DTT pathways allow bypassing DNA lesions during DNA replication to prevent stalling of the replication machinery, since this can lead to greater damages including chromosomal rearrangements. There are two main DDT pathways which may be favored depending, on the type of DNA lesions and phase of the cell cycle [7, 8]. One pathway relies on the recruitment of mostly low-fidelity DNA polymerases to allow incorporation of nucleotides through the lesion, a process referred to as translesion synthesis (TLS). While some polymerases are able to add the correct nucleotides across specific DNA lesions, this process tends to be error-prone [9, 10]. The alternative pathway, which is considered error-free, relies on a template switching (TS) mechanism [11].

In yeast, ubiquitination of Proliferation Cell Nuclear Antigen (PCNA) by the Rad6 E2-conjugating enzyme plays a central role in regulating DDT pathways. Upon replication fork stalling at a DNA damage site, Rad6 together with the Rad18 E3 ligase catalyze PCNA monoubiquitination (at residue K164). This modification promotes the recruitment of TLS polymerases to allow DNA lesion bypass [12, 13]. Alternatively, monoubiquitination can serve as a primer for further polyubiquitination by the Rad5 E3 ligase associated with the methyl methane sulfonate sensitivity-related protein 2 (Mms2)-ubiquitin-conjugating enzyme E2 13 (Ubc13) complex. This complex adds K63-linked ubiquitin chains to promote TS repair mechanisms [12]. Likewise, the mammalian RAD5 homologs, helicase-like transcription factor (HLTF) and SHPRH have been shown to suppress mutagenesis by catalyzing PCNA ubiquitination [7, 14–19].

To date, the study of SHPRH’s enzymatic properties has been limited to PCNA ubiquitination. However, the chromatin-binding domains found in SHPRH suggest that its activity is tightly linked to chromatin. Therefore, we investigated its biochemical properties in the presence of nucleosomal substrates to gain molecular insights into its functions. In this study, we find that SHPRH binds equally well to double-stranded (ds) DNA and to nucleosome core particles, however, SHPRH shows a strong preference for nucleosomes presenting extranucleosomal DNA. While nucleosome binding stimulates the ATPase activity of SHPRH, this stimulation does not however translate into canonical nucleosome remodeling, in standard assays. Furthermore, we tested 26 E2-conjugating enzymes for their potential ability to catalyze histone or nucleosome ubiquitination in the presence of SHPRH. We find that SHPRH is a very efficient histone/nucleosome E3-ubiquitin-ligase which can function with UBE2D- and UBE2E-family, as well as UBE2W conjugating enzymes. Moreover, we show that SHPRH can recruit E2s such as UBE2D1 to nucleosomes and form stable complexes. Mass spectrometry analyses indicated that UBE2D1–SHPRH ubiquitinate nucleosomes with broad specificity and generate diverse or heterogeneous polyubiquitin chains, some of which might serve as recruitment signals for DNA repair factors or for targeting the ubiquitinated proteins to proteasomal degradation. Finally, we uncover that SHPRH is capable of self-ubiquitination. Self-ubiquitination occurs, in part, within functional protein domains hinting to a possible mechanism for autoregulation. Altogether, our data establish a basis for studying the biochemical functions of SHPRH, as well as other RAD5- and potentially other RAD16-related proteins, through the ubiquitination of nucleosomal histones in vitro and in vivo.

## Results

### SHPRH is a dsDNA and a nucleosome-binding protein

In order to characterize SHPRH biochemically, we first cloned a cDNA encoding for the full-length protein from a human transcriptome mRNA library. We then expressed the protein using the baculovirus system and purified it, to apparent homogeneity, via its C-terminal FLAG-tag (Fig. 1a, b, lane 1). Since the SHPRH protein sequence presents domains found in DNA repair and, more generally, in chromatin proteins, we first tested whether it binds to double-stranded (ds) DNA and to nucleosomes.

To this end, we carried out electrophoretic mobility shift assays (EMSAs). As binding substrates, we used DNAs of 147 or 227 base pairs (bp; Fig. 1c, lanes 1 and 9, respectively), or the same DNA fragments assembled into nucleosomes using histones purified from HeLa cells (Fig. 1c, lanes 5 and 13, respectively). Addition of increasing amounts of SHPRH to either the 147 or the 227-bp DNA fragment mainly resulted in large complexes which barely enter the gel, indicating that multiple SHPRH polypeptides bound to these double-stranded DNAs (compare lane 1 (147-bp DNA only) to lanes 2–4 and compare lane 9 (227-bp DNA only) to lanes 10–12). Addition of SHPRH to nucleosome core particles (NCPs) that were assembled using the same 147-bp DNA, resulted in complexes which entered the gel (see diffuse signals at the top of lanes 7 and 8), suggesting that less SHPRH molecules were bound per NCPs compared to free DNA fragments. However, in these conditions, binding was not stable since it did not result in distinct bands. In contrast, addition of SHPRH to a 227-bp DNA which was assembled into mononucleosomes (i.e., 147-bp nucleosome core particles + 80-bp of DNA protruding from the core ‘Nuc + 80’) led to the formation of distinct SHPRH complexes (Fig. 1c lanes 14 and 15). Quantification of these EMSAs showed that the SHPRH binding curve to free DNA is comparable to that of NCPs while binding to mononucleosomes (= NCPs + extranucleosomal DNA) occurred at much lower SHPRH concentrations (Fig. 1d). These results indicate that, similar to ISWI- and CHD-family remodeling factors [20], SHPRH binding to nucleosomes is stabilized by extranucleosomal DNA. These data also hint that SHPRH might preferentially act on nucleosomes during the DNA repair process or during transcriptional regulation.

### SHPRH is a nucleosome-stimulated but not a dsDNA-stimulated ATPase

**Fig. 2:**
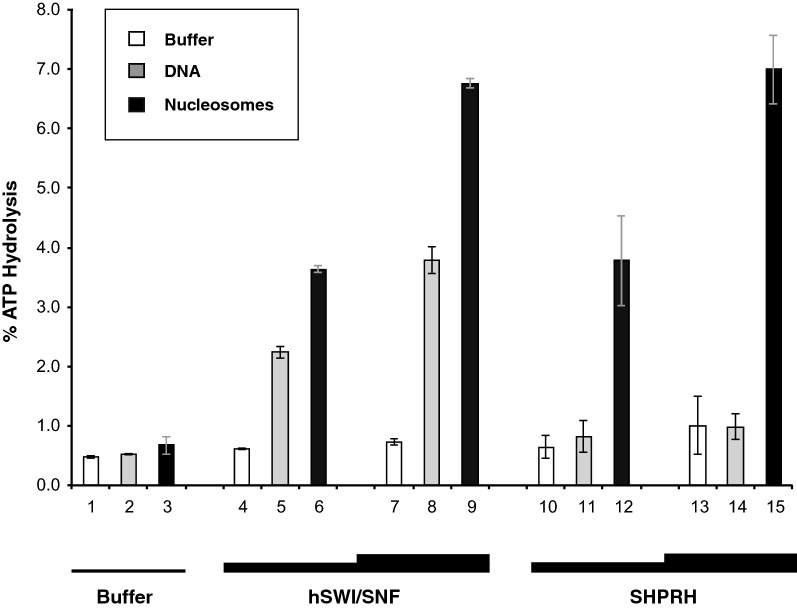
SHPRH is a nucleosome-stimulated but not a dsDNA-stimulated ATPase. ATPase assay. Increasing concentrations of enzymes (hSWI/SNF: 465 ng and 930 ng in a final volume of 15 μl or SHPRH: 57 nM and 170 nM) were incubated in the presence of buffer only (white bars), 5 kb plasmid DNA (200 ng; gray bars) or the same plasmid assembled into nucleosome arrays (200 ng; black bars) and 20 μM ATP/60 μM Mg^2+^ supplemented with traces of γ-[32P]ATP. Hydrolyzed phosphates and non-hydrolyzed ATP were separated using thin layer chromatography after incubating the reactions for 40 min at 37 °C. The percentage of ATP hydrolysis was quantified using a phosphorimager. Error bars reflect standard deviation derived from two independent experiments

Since SHPRH binds to both DNA and nucleosomes and SHPRH harbors a (non-canonical) SNF2-family ATPase domain (Fig. 1a), we asked whether, like other SNF2-family enzymes, SHPRH is a DNA and/or a nucleosome-stimulated ATPase. We therefore incubated recombinant SHPRH in the presence of buffer only (Fig. 2 white bars 10 and 13), plasmid DNA (gray bars 11 and 14) or the same plasmid assembled into an array of nucleosomes (black bars 12 and 15). As a control ATPase, we used the well-characterized human (h) SWI/SNF chromatin remodeling complex (Fig. 1b, lane 3). As previously shown (for yeast SWI/SNF), addition of dsDNA or nucleosomes to hSWI/SNF resulted in a strong ATPase activity stimulation (about 3.6 to 5-fold or ~ 6 to ~ 9-fold increase, respectively). In contrast to hSWI/SNF, and consistent with previous data obtained using the related yeast RAD5 protein [21], the ATPase activity of SHPRH was not stimulated by addition of dsDNA to the reactions. However, addition of nucleosome arrays resulted in a strong ATP-hydrolysis stimulation (~ 6 to ~ 7-fold increase). These results further support the idea that, in the context of DNA repair or transcriptional regulation, SHPRH might preferentially act on nucleosomes (and/or possibly on particular DNA structures, see discussion).

### SHPRH does not display nucleosome remodeling activity in standard remodeling assay conditions

**Fig. 3:**
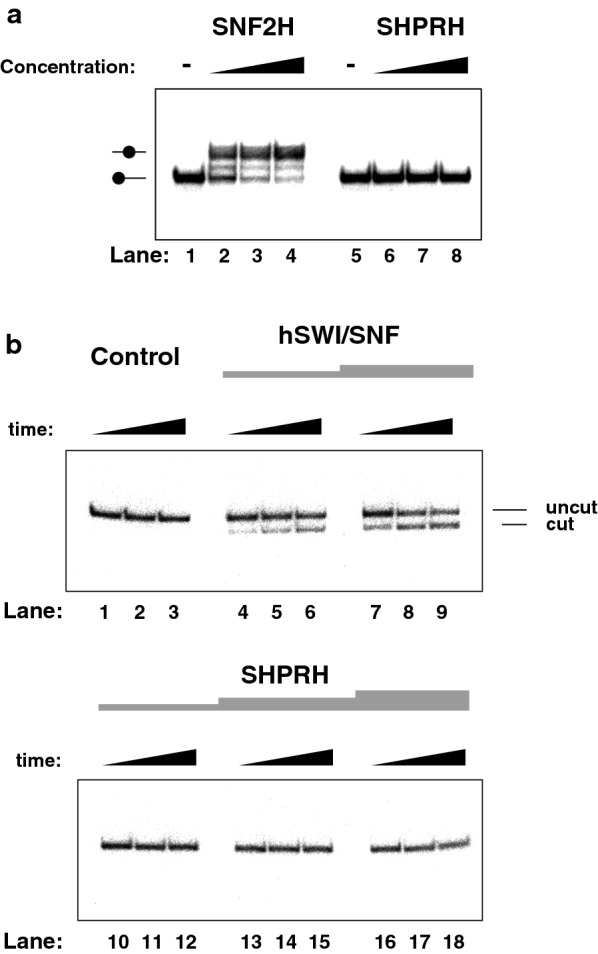
SHPRH does not display conventional nucleosome remodeling activity. **a** Nucleosome mobilization assay. Increasing concentrations of enzymes (SNF2H: 13 nM, 26 nM, 52 nM or SHPRH: 43 nM, 85 nM, 170 nM) were incubated with 20 nM of radiolabeled end-positioned nucleosome comprising an 80-bp DNA overhang. Migration of the end- or the centrally positioned-nucleosome (filled circle) are indicated on the left. **b** Analysis of nucleosome remodeling activities by restriction enzyme accessibility (REA) assay. The assay measured the ability of the tested enzymes to expose an MfeI restriction site in the nucleosome at + 28-bp. Control (buffer only), remodeling factors (hSWI/SNF 93 ng and 186 ng in 20 μl; SHPRH 17 nM, 34 nM and 68 nM) were incubated with (20 nM) radiolabeled nucleosomes with a 80-bp DNA overhang, in the presence of ATP (2 mM) and the reaction were stopped at 20, 40 and 60 min

SNF2-family proteins typically use ATP hydrolysis to rearrange histone-DNA contacts, a process referred to as nucleosome or chromatin ‘remodeling.’ We therefore examined whether the increase in ATP hydrolysis triggered by the presence of nucleosomes (seen above), reflected the ability of SHPRH to remodel nucleosomes. To test this, we used the standard ‘nucleosome sliding’ (or ‘nucleosome mobilization’) assay. This approach takes advantage of the correlation between nucleosome positions on a DNA fragment and their electrophoretic mobility in native PAGE. For instance, nucleosomes positioned at the end of a DNA fragment migrate faster than nucleosomes positioned in the middle of the same DNA fragment. Hence, ATP-dependent nucleosome repositioning by chromatin remodeling factors can be monitored by observing changes in electrophoretic mobility (after the remodeling factor is competed off the nucleosomes using unlabeled DNA). As expected, such changes in electrophoretic mobility could be observed upon repositioning of the histone octamer from the end of the 227-bp DNA to a more central position, when the SNF2H-nucleosome remodeling factor (Fig. 1b, lane 2) and ATP were added to the mononucleosome (Fig. 3a compare lane 1, nucleosome only, to lanes 2–4, nucleosome with increasing concentrations of SNF2H). Surprisingly for a SNF2-family protein, SHPRH was inactive, in this assay (Fig. 3a lanes 6–8).

Given that SHPRH could potentially reposition nucleosomes to the other end of the DNA fragment (or back to its original location) and that, as seen with SWI/SNF-subfamily remodelers [22], this would not result in detectable nucleosome mobility shifts, we switched to using the restriction enzyme accessibility (REA) assay. In this assay, DNA is protected from restriction enzyme cleavage when it is wrapped around the histone octamer (Fig. 3b, lanes 1–3; [23]). When the histones are moved away from the restriction site by a nucleosome remodeling factor, DNA gets cleaved and after deproteinization DNA cleavage is assessed by PAGE. Thus, nucleosome remodeling is inferred from DNA cleavage. Addition of a nucleosome remodeling factor such as hSWI/SNF to a mononucleosome leads to repositioning of the histones and consequently cutting of the exposed DNA (Fig. 3b, lanes 4–9). Consistent with the sliding assay results, addition of SHPRH to a mononucleosome did not lead to any increase in DNA accessibility, over a wide range of concentrations (lanes 10 to 18). Collectively, these data suggest that unlike most SNF2-family proteins, SHPRH is not a classical nucleosome remodeling factor, at least under standard assay conditions.

### Screen for E2-conjugating enzymes that catalyze histone/nucleosome ubiquitination with SHPRH

**Fig. 4:**
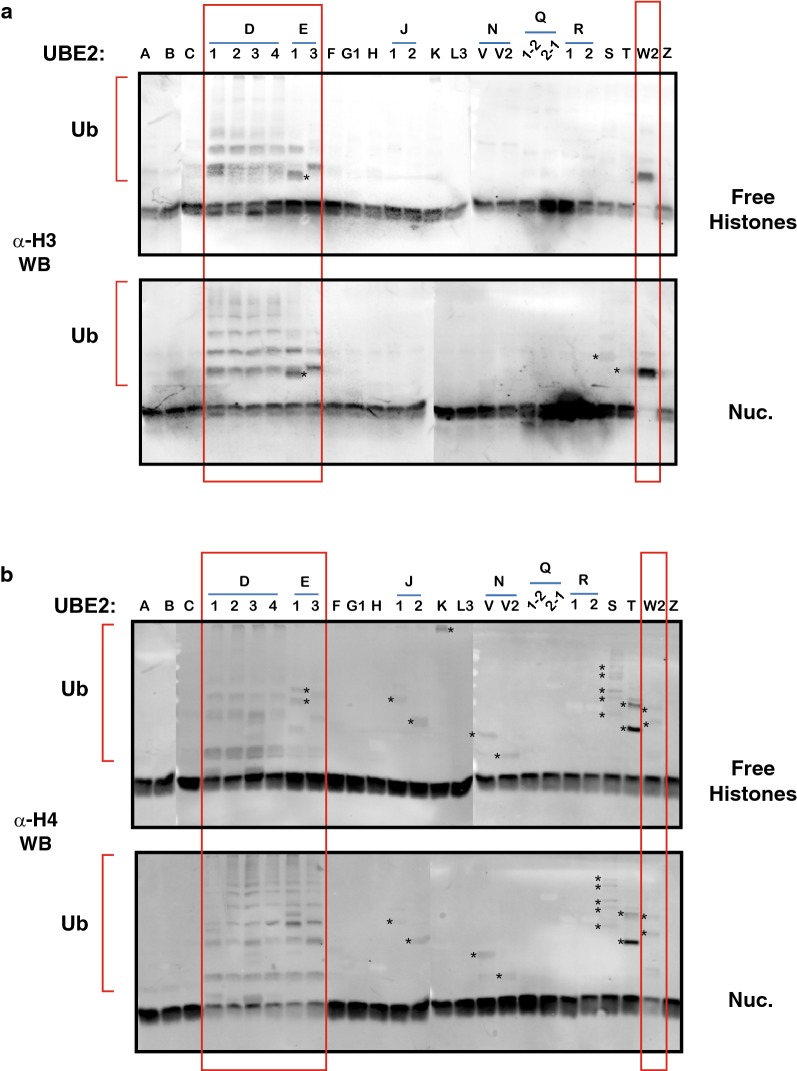
SHPRH is a histone/nucleosome E3-ligase. **a** E2-conjugating enzyme screen. Twenty-six different E2s (as indicated on top of the panels) were tested for their ability to support free or nucleosomal histone ubiquitination by the SHPRH E3-ligase. Histone octamers purified from HeLa cells (upper panel) or nucleosome arrays (lower panel) were used as substrates for ubiquitination. SHPRH (85 nM) and the substrates (600 ng histones or nucleosome arrays per 20 μl reaction) were added to a mix containing E1, E2-conjugating enzymes and ubiquitin, and the reactions were incubated for 1 h at 37 °C prior to adding SDS loading buffer to stop the reactions. Samples were then boiled and the proteins were separated on a 15% PAA gel followed by Western blotting against histone H3. **b** the same samples as in (**a**) were blotted using an antibody against histone H4. (Parts of gels from the same experiment were aligned using Photoshop for clarity). Asterisks denote non-specific Western Blot signals (based on the Coomassie-stained E2 gel provided by Boston Biochem)

Previous studies have shown that, when the DNA replication machinery encounters a DNA damage and stalls, RAD5-related proteins such as HLTF and SHPRH interact with MMS2/Ubc13 (UBE2V2/UBE2N) E2-ubiquitin-conjugating enzyme to catalyze PCNA polyubiquitination [14–18]. Since SHPRH stably interacts with nucleosomes, this prompted us to test whether SHPRH can also catalyze the ubiquitination of free or nucleosomal histones. To determine which E2 might act as a ubiquitin donor in this reaction, we tested a set of 26 different E2 enzymes which belong to all 4 major classes of E2s. The reactions were then subjected to SDS PAGE and we assessed histone ubiquitination by Western Blot, using antibodies against all 4 core histones. Despite testing 2 different antibodies, the results for H2A were not conclusive. Similarly, H2B ubiquitination analyses were only partially informative as most Western Blot signals were too weak. In contrast, detection of H3 and H4 ubiquitination was robust. As shown in Fig. 4, seven different E2s catalyzed ubiquitination of free or nucleosomal histone H3 (Fig. 4a, lanes within frames, upper panel: free histones; lower panel: nucleosomes) and the same E2s also carried out free or nucleosomal H4 ubiquitination (Fig. 4b, lanes within frames, upper and lower panel, respectively). First, all four members of the UBE2D family (Lanes D1 to D4, formerly UBCH5 family, members A to D, respectively) were very efficient at promoting both free and nucleosomal H3 and H4 polyubiquitination, in the presence of SHPRH. In addition, two members of the UBE2E-family and UBE2W2 were also efficient at catalyzing ubiquitination. For clarity, we analyze these results separately below.

### UBE2E-family E2s catalyze SHPRH-directed monoubiquitination of histone H3 residues and nucleosome-specific polyubiquitination of H4

In the presence of UBE2E1 (UbcH6), we observed predominantly mono- and di-ubiquitination of both free and nucleosomal H3 (as judged by the protein migration). Since an N-terminal extension of UBE2E enzymes generally limits the formation of polyubiquitin chains [24], it is likely that, when SHPRH associates with UBE2E1, it catalyzes the monoubiquitination of two different residues on H3 (Fig. 4a upper and lower panel). Surprisingly, in contrast to what we observed with H3, UBE2E1 promoted H4 polyubiquitination and the reaction was also highly nucleosome-specific. Indeed, while barely any H4 was modified in reactions containing free histones, most of H4 was remarkably polyubiquitinated when nucleosomes were used as a substrate (Fig. 4b compare upper to lower panel, lane E1). This observation was unexpected given the above-mentioned and established role of the N-terminus of Class III E2s in preventing such polyubiquitination reactions. This implies that, when UBE2E1 is positioned over H4, a nucleosomal epitope relieves the N-terminal inhibition of the ubiquitin-conjugating (UBC) domain. Alternatively, when bound to nucleosomes, SHPRH induces a conformational change of UBE2E1 which allows H4 polyubiquitination. These possibilities are not mutually exclusive. Similarly, UBE2E3 (UbcH9/UbcM2) mainly promoted monoubiquitination of free H3 and the reaction was more efficient on nucleosomal H3. The appearance of a di-ubiquitinated form of H3 also suggests that this additional ubiquitination of a particular residue is nucleosome-specific. Strikingly and similar to UBE2E1, UBE2E3 did not support ubiquitination of free H4, whereas nucleosomal H4 was very efficiently polyubiquitinated. This suggests that again, upon binding to nucleosomes, SHPRH–UBE2E complexes adopt a catalytically favorable conformation which allows histone H4 polyubiquitination.

### UBE2W2 promotes N-terminal monoubiquitination of H3 and nucleosomal H4

When using the isoform 2 of UBE2W (UBC16) as a ubiquitin-conjugating enzyme, monoubiquitination reactions of free and nucleosomal H3 were very efficient and went to completion, in these conditions. Interestingly, SHPRH–UBE2W also showed a very weak, but noticeable, activity on nucleosomal H4 but not on free H4 (note the reduction in signal intensity for unmodified nucleosomal H4; Fig. 4b lower panel). UBE2W has been shown to modify the α-amino group of disordered protein N-termini, independently of substrate protein sequence [25–27]. These results are therefore consistent with the idea that histone N-terminal tails are mainly disordered [28].

### UBE2D–SHPRH complexes catalyze broad ubiquitination of all nucleosomal core histones

As mentioned above, all four members of the UBE2D family were very efficient at promoting both free and nucleosomal H3 and H4 polyubiquitination. Given the prominence of this family in robustly supporting nucleosome ubiquitination by SHPRH and given the established role of this E2 family in both nucleosome ubiquitination (by Polycomb-group proteins [29, 30]) and in DNA repair [31], we focused on analyzing the products of the ubiquitination reactions catalyzed by the representative member UBE2D1, by mass spectrometry. As substrates, we used nucleosomes that were reconstituted with recombinant histones. First, these analyses confirmed H3 and H4 ubiquitination (seen in Fig. 4) and, in addition, revealed that H2A and H2B were also ubiquitinated. The variability of the modification sites between independent experiments suggests that SHPRH–UBE2D1 show broad specificity, in these conditions (Additional file 1). These observations further support the hypothesis that these E2s function with most E3-ligases and carry out most of the ubiquitination events in the cell [32].

### SHPRH–UBE2D1 modify all the 7 lysines of ubiquitin, in vitro

Polyubiquitin chains often carry out their function through factors which recognize the type of linkage between their ubiquitin moieties rather than being functionally specific for the targeted protein residue(s) [33]. Therefore, we analyzed which of the ubiquitin residues were modified by addition of other ubiquitin moieties, as an indication of which polyubiquitin chains may be generated by SHPRH–UBE2D1. We find that all the 7 ubiquitin lysines (K6, K11, K27, K29, K33, K48 and K63) were modified, suggesting that again the E2–E3 complex showed relaxed enzymatic specificity (Additional file 2). Given this diversity of modifications, our analysis did not allow us to distinguish if homogeneous, mixed or branched ubiquitin chains were formed. However, UBE2D1 has previously been shown to have an intrinsic preference for forming polyubiquitin chains through K11, K48 and K63 [34]. If this preference is confirmed when associated with SHPRH in vivo, it would imply that SHPRH mediates the formation of polyubiquitin chains that are associated with DNA damage-induced transcriptional silencing, DNA repair (K11 and K63, respectively [35]) and proteasomal degradation (K48 [33]).

### SHPRH stably recruits UBE2D1 to nucleosomes

**Fig. 5:**
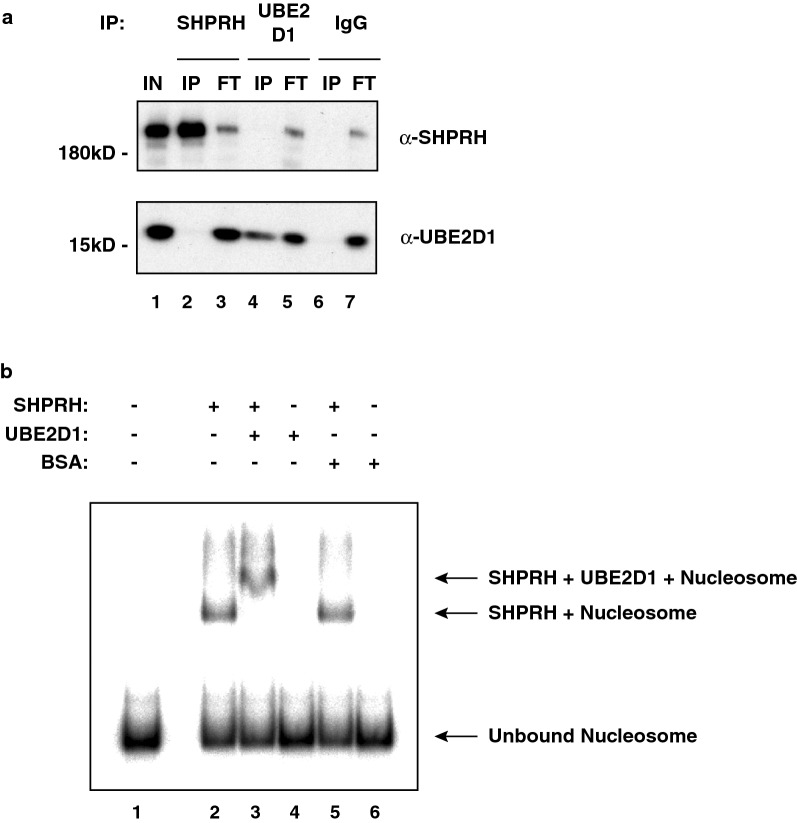
SHPRH recruits UBE2D1 to, and forms stable complexes with, nucleosomes. **a** Western blot of SHPRH and UBE2D1 immunoprecipitations. FLAG-tagged versions of SHPRH and UBE2D1 were overexpressed in HEK293 cells. Whole cell extracts were then used to immunoprecipitate the two proteins using specific antibodies, as indicated on top. IgG was used as a negative control. The input (IN), the flow-through (FT) and the immunoprecipitates (IP) were probed using antibodies (α) against SHPRH and UBE2D1, as indicated on the right. **b** Electrophoretic mobility shift assay. SHPRH (20 nM) was incubated with radiolabeled mononucleosomes (20 nM; lane 2), or, in addition, with UBE2D1 (800 nM; lane 3). As controls, we used mononucleosomes alone (20 nM; lane 1) or incubated in the presence of UBE2D1 or BSA (800 nM; lanes 4 and 6, respectively) or, SHPRH + BSA (lane 5), as indicated on top. Reactions were separated by native PAGE and visualized using a phosphorimager screen. The positions of free (unbound) nucleosomes and protein-nucleosome complexes are indicated by arrows, on the right

To investigate the potential relevance of our in vitro data further, we tested whether SHPRH and UBE2D1 interact in cell extracts. Since most E2–E3 complexes are very unstable [36], we overexpressed both SHPRH and UBE2D1, in HEK293 cells, prior to performing immunoprecipitations. When using an antibody against SHPRH, we immunoprecipitated the protein very efficiently. However, we did not enrich for UBE2D1 in the precipitated material (Fig. 5a, lane 2). Similarly, immunoprecipitation of UBE2D1 did not co-precipitate SHPRH (lane 4). Given that we observed nucleosome ubiquitination in our assays above, this prompted us to test whether the E2–E3 interaction might be dependent on the presence of the nucleosome substrate. To this end, we performed EMSAs. As shown in Fig. 1c, addition of SHPRH to nucleosomes leads to the formation of complexes which migrate slower (Fig. 5b; compare nucleosome without SHPRH lane 1 to nucleosome with SHPRH lane 2). In contrast, addition of UBE2D1 or BSA to the nucleosome substrate did not result in binding, as judged by the absence of any noticeable shift (lanes 4 and 6, respectively). However, addition of UBE2D1 to SHPRH nucleosome complexes resulted in a supershift, indicating that SHPRH can recruit UBE2D1 to nucleosomes (compare lane 2 to lane 3). The supershift was specific since, as a control, addition of BSA to SHPRH nucleosome complexes did not result in the formation of a supershift. These data demonstrate that SHPRH and UBE2D1 can form stable complexes with nucleosomes and support the idea that this E2-E3 interaction might be reinforced by nucleosomes.

### SHPRH catalyzes self-ubiquitination in the presence of UBE2D1

Most E3 ligases have the ability to catalyze their own ubiquitination [37]. We therefore investigated whether, in the presence of UBE2D1, SHPRH also shares this ability. Using mass spectrometry, we find that indeed SHPRH can ubiquitinate itself at many sites. Interestingly, the modifications can be found within key motifs of the SNF2-helicase domain (e.g., K905 and K906 within Motif IV; Additional file 3) and many of the modifications have been identified in vivo (e.g., K1062, K1256, K1562, K1572 *and* K1647; see database section below for references). Given their critical location, we think it likely that some of these modifications affect the activity of SHPRH. Therefore, these modifications warrant further investigations to determine their impact on the functions of SHPRH, in vitro and in vivo.

### Discussion

In summary, we present the first biochemical characterization of the SNF2-family protein SHPRH, in the presence of nucleosome substrates, in vitro. We find that SHPRH is a nucleosome-stimulated ATPase, however, unlike most SNF2-family members, SHPRH does not appear to translate ATPase activity into canonical nucleosome remodeling, under standard assay conditions. Given SHPRH’s preference for binding mononucleosomes over dsDNA and its ability to catalyze PCNA ubiquitination, we explored the possibility that SHPRH ubiquitinates nucleosomes. We show that indeed SHPRH is a potent histone/nucleosome E3-ligase which can function with class I and class II E2-conjugating enzymes, in vitro. Furthermore, we provide data, suggesting that, in some instances, ubiquitination reactions are nucleosome specific. Finally, we also provide evidence that SHPRH can initiate monoubiquitination (in addition to the already known ability to catalyze polyubiquitination), and that this enzyme can also perform self-ubiquitination.

#### SHPRH substrate preferences

We find that SHPRH preferentially binds to mononucleosomes compared to free dsDNA and that its ATPase activity is stimulated by nucleosomes but not by dsDNA. It is possible that SHPRH has a preference for binding DNA substrates which were not tested in our study. For instance, SWI/SNF-related, matrix-associated, actin-dependent regulator of chromatin, subfamily a-like 1 (SMARCAL1/HARP), zinc finger RANBP2-type containing 3 (ZRANB3) and RAD5 proteins prefer binding to structures with a single-stranded/DNA-double-stranded DNA junction [38–42]. Likewise, the ATPase activity of SHPRH might also be stimulated by complex DNA structures such as Y fork or four-way junction DNAs, as seen for the related RAD5 protein [21]. These possibilities remain to be tested. Yet, the fact that SHPRH harbors chromatin-binding domains and that nucleosomes trigger ATP hydrolysis strongly indicates that SHPRH carries out its function, at least in part, on nucleosomes. This notion is also consistent with recent data from the Myung laboratory uncovering that SHPRH also functions outside of DNA repair, namely in the regulation of rDNA transcription [43].

#### SHPRH does not appear to remodel nucleosomes in conventional remodeling assays

Despite testing several protein preparations, assessing a wide range of protein concentrations, using different nucleosome substrates or incubating the nucleosome remodeling reactions for extended periods of time, we were unable to detect any nucleosome remodeling activity by SHPRH, under standard assay conditions. This was surprising since SNF2-family nucleosome-stimulated ATPases typically translate ATP hydrolysis into nucleosomal DNA translocation and, consequently, alter DNA accessibility [44]. It is possible that SHPRH purified from Sf9 cells is not properly folded. However, we think it unlikely since the protein is active in 3 different biochemical assays, namely nucleosome binding, ATPase and ubiquitin E3-ligase assays. It is also possible that, as seen for the DNA repair protein Cockayne syndrome B (CSB also referred to as excision repair cross-complementation group 6 (ERCC6) [45, 46]) or the DNA methylation regulator lymphoid-specific helicase (LSH/HELLS [47]), SHPRH requires a protein cofactor to perform the remodeling reaction (or possibly the presence of a specific E2). It would therefore be interesting to determine whether SHPRH is actually part of a protein complex, in vivo.

Alternatively, SHPRH might only remodel specific substrates (e.g., nucleosomes with a particular histone modification, histone variant or the presence of histone H1 since SHPRH harbors an H1-like domain). The observation that SHPRH binds better to nucleosomes with extranucleosomal DNA might also indicate that SHPRH might remodel nucleosomes that are adjacent to specific DNA structures found at stalled replication forks. For instance, remodeling might require nucleosomes to be flanked by a so-called Holliday junction DNA structure which can be observed during replication fork regression. This supposition would be consistent with the presence of a weak homology to the RuvC junction-resolving enzyme (NCBI conserved domain database E value = 5.2) between the TPR and RING finger domains of SHPRH (Fig. 1a).

In addition, SHPRH remodeling activity might be triggered by post-synthetic modification(s) which might occur during DNA repair or cell starvation (see below). All the possibilities mentioned above are not mutually exclusive or could also be required, in combinations. Further studies will be required to address these points.

#### SHPRH and ubiquitination

In a screen for E2s which might support histone/nucleosome ubiquitination by SHPRH, we identify 7 different proteins which can mediate mono- and/or polyubiquitination, in vitro. Importantly, the fact that SHPRH functionally interacted with only a subset of E2s supports the idea that the reactions were specific. Of note, the MMS2/Ubc13 (UBE2V2/UBE2N) complex, which is known to associate with SHPRH to catalyze PCNA polyubiquitination, did not display activity on (nucleosomal) histones. However, this complex does not initiate PCNA monoubiquitination. It is therefore still possible that UBE2V2/UBE2N and SHPRH might function together on monoubiquitinated (“primed”) nucleosomes and catalyze their polyubiquitination, as seen for RNF8 [48]. For instance, we find that SHPRH–UBE2W can catalyze N-terminal monoubiquitination and this modification can be extended into polyubiquitin by the MMS2/Ubc13 complex [49]. Moreover, most linkage-specific chain-building E2s require their substrate to be primed prior to catalyzing polyubiquitination [36]. Therefore, by extension, other E2s in this screen might only work in concert with SHPRH if nucleosomes are first monoubiquitinated.

In contrast to these E2s, all four members of the UBE2D family were very efficient at promoting both free and nucleosomal H3 and H4 polyubiquitination. These results are consistent with the fact that all the members of this family share at least 92% of protein sequence identity [50]. UBE2D proteins often display broad specificity ([36, 51] and refs. therein), and these enzymes have been hypothesized to assist most ubiquitination reactions in human cells [32]. Thus, these E2s might be enzymatically advantageous in the context of DNA repair, since the protein composition of repair sites is highly variable, including the post-translational modification state of potential target lysine residues.

#### UBE2E1/3–SHPRH complexes catalyze more restricted and nucleosome-specific modifications

The activity of UBE2E-conjugating enzymes clearly contrasted with that of UBE2D proteins. Indeed, these enzymes were unable to drive efficient H3 polyubiquitination. Instead, UBE2E3 and UBE2E1 appear to catalyze the addition of 1 or 2 ubiquitination moieties to histone H3, respectively. The limited number of target lysines may reflect a high degree of specificity of these E2-SHPRH complexes. This behavior is reminiscent of the activity of UBE2E1 which was shown to be critical for the specific H2AK119 monoubiquitination by the transcriptional repressor PRC1, in vivo [52]. While on H3, the activity of UBE2E1/3–SHPRH complexes was most likely limited to monoubiquitination events, histone H4 was not only polyubiquitinated, but the reaction was nucleosome specific. Given that UBE2E3 can synthesize K63-linked poly-Ub chains (which promote template switching DNA repair mechanisms; [53]), it is tempting to speculate that UBE2Es–SHPRH might synergize in the context of DNA repair, in vivo. In addition, our in vitro results raise the possibility that SHPRH has different nucleosome-binding modes.

#### SHPRH catalyzes self-ubiquitination in the presence of UBE2D1

We find that SHPRH catalyzes its own ubiquitination in the presence of UBE2D1. Our data are consistent with whole proteome ubiquitination studies which have shown that SHPRH is ubiquitinated in vivo and most of the target we identified overlap with these in vivo sites (see database section below). Our findings are also consistent with previous studies showing that most RING finger-types of E3s catalyze self-ubiquitination. This modification often serves as a regulatory mechanism which triggers degradation [37, 54, 55]. However, as seen for other E3s (or for other post-synthetic modifications), ubiquitination might also regulate SHPRH’s biochemical activities, namely ATPase, nucleosome binding and/or E3 ligase activities. It is intriguing that some of these modifications are found at residues within key motifs of the SNF2 catalytic domain or at insertions within this domain. Interestingly, the histone methyltransferase SET domain bifurcated 1 (SETDB1) requires to be ubiquitinated, at a residue (K867) within an insertion found in its catalytic SET domain, to be active. The modification was shown to be essential for enzymatic activity and for its function, in vivo [56]. Similarly, ubiquitination of the more closely related CSB ATPase is also required for functions including RNA polymerase II recruitment after UV irradiation [57]. These studies therefore raise the question whether ubiquitination of SHPRH renders it competent for nucleosome remodeling or other functions.

#### Histone ubiquitination and DNA repair

Many studies have established that protein ubiquitylation, in general, and histone ubiquitylation, in particular, play a prominent role in DNA repair ([58–60] and refs. therein). All four core histones, and the linker histone H1, have been shown to be ubiquitinated in response to various genomic insults [61–64]. Ubiquitylation of histones (and other proteins) by SHPRH might serve as a signal for targeting the proteins to degradation, such that DNA repair factors may gain better access to DNA [61, 65–68]. Additionally, ‘stripping’ off chromatin bound factors and chromatin disassembly (resulting from the action of SHPRH) might promote replication fork reversal [69]. Ubiquitination by SHPRH might also function as a recruitment signal for repair factors and/or might exclude proteins from binding chromatin [70]. Modulation of chromatin binding may consequently contribute to driving cells down specific DNA repair pathways. For instance, different histone ubiquitination marks have been shown to promote different repair mechanisms such as homologous recombination or non-homologous end joining. Given the implications of H2B monoubiquitination in DNA damage bypass after UV damage or MMS treatment [71, 72], it remains to be established whether, in addition to PCNA ubiquitylation, histone ubiquitylation by SHPRH might also contribute to the choice between different arms of the DNA damage tolerance pathways. Likewise, it would be interesting to test whether, despite their lack of obvious histone binding domains, RAD5- or RAD16-related proteins including HLTF, the yeast proteins increased recombination centers 20 (IRC20/YLR247C), or as hypothesized previously by Yu et al., ubiquitin ligase for SUMO conjugates protein 1 (ULS1/role in silencing protein 1 (RIS1); [73]) can ubiquitinate nucleosomes directly, or once they are primed by monoubiquitination. Lastly, such studies might also be extended to other ubiquitin-like modifiers such as SUMO.

## Conclusion

The study of SHPRH enzymatic functions had so far been focused on the polyubiquitination of PCNA, at stalled replication forks. Our results expand the scope of SHPRH activities beyond this modification. Although the in vivo relevance of these observations will require further investigations, several general principles can be derived from our study. We uncovered that SHPRH prefers binding to mononucleosomes over dsDNA, that nucleosomes stimulate its ATPase activity and that SHPRH can function with different E2-conjugating enzymes to catalyze nucleosome ubiquitination. Collectively, these data lay the ground work for investigating the functions of SHPRH with a focus on nucleosome-based activities, in the context of DNA repair as well as the regulation of transcription, or in yet unanticipated pathways, in vivo. In addition, our biochemical characterization might have more general implications in DNA repair as it raises the possibility that other RAD5- or RAD16-related proteins might also carry out their function, in part, through nucleosome ubiquitination.

## Materials and methods

### Cloning

The SHPRH cDNA (NCBI NM_001042683.3) was cloned from MegaMan Human Transcriptome Library (Agilent Technologies), and a C-terminal FLAG-tag was introduced upstream the STOP codon by PCR. Mutations were corrected using the QuikChange Site-Directed Mutagenesis kit according to the manufacturer’s instructions.

### Protein expression and purification

Protein expression and purification (and other methods below) were essentially carried out as described previously in [20]. Briefly, SHPRH baculoviruses were produced according to the Bac-to-Bac Baculovirus Expression Systems manual (Life Technologies). SHPRH-FLAG protein preparations were obtained from 4L Sf9 cell cultures. Cells were resuspended in BC buffer [10% Glycerol, 20 mM HEPES, pH 7.9, 0.4 mM EDTA, freshly supplemented with β-mercaptoethanol and protease inhibitors] containing 250 mM NaCl (BC 250). Cells were lysed by 2 freeze–thaw cycles, and the cell extracts were cleared by centrifugation before adding M2-affinity (anti-FLAG) gel (Sigma) for 4 h, at 4 °C. The extract containing the affinity gel was poured into Econo columns (Bio-Rad), and the flow-through was reapplied once. The beads were then washed twice with at least 10 resin volumes of: BC 250, BC 500, BC 1000 and once with about 10 resin volumes of BC 500, BC 250 and finally with 10 resin volumes of BC 100 prior to elution with BC 100 containing 0.25 mg/ml of FLAG-peptide (Sigma).

### DNA and nucleosome substrate preparations

DNA fragments (of 147 or 227 bp) used for nucleosome assemblies and bandshift assays were generated using the ‘601’ nucleosome-positioning sequence (Lowary PT and Widom [74]). All DNAs were amplified by PCR including radiolabeled α-[32P]-dCTP. After purification, the DNAs were assembled into nucleosomes by standard salt dialysis using histones purified from HeLa cells or recombinant histones produced in bacteria. After reconstitution, nucleosomes were purified using 10–30% (vol/vol) glycerol gradients, for 18 h at 35,000 rpm using a Beckman SW55Ti rotor.

### Nucleosome electrophoretic mobility shift assay (EMSA)

The nucleosome EMSAs were carried out using 20 nM of radiolabeled DNA or nucleosomes (as indicated in the figures), in 12 mM HEPES pH 7.9, 10 mM TRIS pH 8.0, 50 mM NaCl, 4 mM MgCl_2_, ~ 6% glycerol, 0.02% IGEPAL^®^ CA-630 (Sigma-Aldrich), 0.15 mM EDTA pH 8.0. Increasing concentrations of SHPRH (57, 113, 170 nM) were added to the substrates and after incubation on ice for 10 min, free and protein-bound substrates were separated by 5% native PAGE, 0.5 × TBE for about 3h30 at 110 V. Gels were dried and exposed to a phosphorimager screens overnight. The screens were then scanned using a Fuji FLA5000 device and quantified with Science Lab Image Gauge (FujiFilm).

### ATPase assays

hSWI/SNF (465 ng and 930 ng) in a final volume of 15 μl or SHPRH (57 nM and 170 nM) was incubated in the presence of buffer only, 5 kb plasmid DNA (200 ng) or the same plasmid assembled into nucleosome arrays (200 ng of DNA equivalent) and 20 μM ATP/60 μM Mg^2+^ supplemented with traces of γ-[32P]ATP. Hydrolyzed phosphates and non-hydrolyzed ATP were separated by thin layer chromatography (Baker-flex’ Cellulose PEI-F or Millipore) using 0.5 M LiCl/1 M formic acid buffer, after incubating the reactions at 37 °C, for 40 min. ATP hydrolysis was detected using a phosphorimager screen, scanned with a Fuji FLA5000 device and quantified using Science Lab Image Gauge (FujiFilm).

### Nucleosome mobilization assay

Reactions were carried out in a final volume of 20 μl. 12 μl BC100 buffer (control) or enzymes (SNF2H: final concentrations of 13 nM, 26 nM, 52 nM or SHPRH: final concentrations of 43 nM, 85 nM, 170 nM) were incubated with 4 μl of glycerol gradient buffer (see above) containing a radiolabeled end-positioned nucleosome (NCP + 80-bp DNA overhang) at a final concentration of 20 nM. 4 μl of a 10 mM ATP/30 mM Mg^2+^ mix was added to start the reactions. An aliquot was taken out after either 20 min or 40 min at 30 °C, and the reactions were stopped by addition 2 μl of a mix containing unlabeled competitor DNA (1.5 μg/μl) and EDTA (125 mM), incubated on ice for 10 min before 5% native PAGE with 0.5 × TBE. Gels were dried, exposed to a phosphorimager and visualized as described above.

### Restriction enzyme accessibility assays

The assay measured the ability of the tested enzymes to expose an MfeI restriction site in the nucleosome at + 28-bp. Control (buffer only), remodeling factors (hSWI/SNF 93 ng and 186 ng in 20 μl; SHPRH 17 nM, 34 nM and 68 nM) were incubated with 20 nM of radiolabeled nucleosomes with a 80-bp DNA overhang, 1.5 U/μl of MfeI-HF (New England Biolabs), in the presence of ATP (2 mM) and the reaction were stopped at 20, 40 and 60 min, and visualized as described above.

### UBE2 screen and ubiquitination assays

The screen for UBE2s which might support histone or nucleosome ubiquitination by SHPRH was carried out using the E2Select Ubiquitin Conjugation Kit from Boston Biochem (K-982). All steps were done according to the manufacturer’s instructions; substrates (histones or polynucleosomes) were added to a final concentration of 30 ng/µl per well (in 20 µl final volume). Additional ubiquitination assays (to confirm our results and for mass spectrometry purposes) were performed in similar conditions, using UBE1, UBE2 and ubiquitin at final concentrations of 50 nM, 25 µM and 50 µM, respectively. All additional materials were purchased from Boston Biochem (Ubiquitin: U-100H; His6-Ubiquitin E1 Enzyme: E-304; Ubc5a: E2-615 and E2-616; Ubc5c: E2-625). Proteins were then separated by SDS PAGE and (ubiquitinated) histones were probed by Western Blot using the following primary antibodies: anti-H4 (New England Biolabs, 2592 S) and anti-H3 (Abcam, ab1791). IRDye secondary antibodies from LiCor (P/N 925-32210, P/N 925-68020) were used for detection using a LiCor Odyssey Imaging System.

### Cell transfections and Immunoprecipitations of SHPRH and UBE2D1

Empty, SHPRH-FLAG and/or UBE2D1-FLAG pcDNA3.3 vectors were co-transfected in HEK293 cells using X-tremeGENE™ HP DNA Transfection Reagent (Sigma-Aldrich, 06366244001) according to the manufacturer’s instructions. Cells were harvested 24 h post-transfection, and whole cell extracts were prepared by resuspending cells in RIPA buffer (50 mM Tris–HCL pH7.8, 150 mM NaCl, 0.2% NP-40, 0.25% sodium deoxycholate, 1 mM EDTA), followed by 3 freeze–thaw cycles. Benzonase (Merck/Millipore, 70664-3) and MgCl_2_ (to a final concentration of 7.5 mM) were added to the cells and incubated for 30 min on ice before centrifuging cell debris. The extracts were then pre-cleared for 2 h using Protein G Sepharose 4 Fast Flow antibody purification resin from GE Healthcare before carrying out immunoprecipitations using primary antibodies against SHPRH (OriGene, TA501443), UBE2D1 (Abcam, ab176561) and a control IgG (Sigma-Aldrich, I5381), for 2 h. The IPs were incubated in the cold room for an additional 2 h after adding Protein G Sepharose resin (50 µl resin was used per 1 µg of antibody), and Western Blot detection of the immunoprecipitates was performed using the same above-mentioned primary antibodies for SHPRH and UBE2D1.

### Analyses by mass spectrometry

Samples bound to (magnetic) beads were washed three times with 100 µL 0.1 M ammonium bicarbonate solution. They were digested ‘on-beads’ by the addition of sequencing grade modified trypsin or ArgC and incubated at 37 °C for 45 min. Subsequently, the supernatant was transferred to fresh tubes. Next, samples were reduced and carbamidomethylated. Peptides were desalted and concentrated using Chromabond C18WP spin columns (Macherey–Nagel, Part No. 730522). Finally, peptides were dissolved in 25 µl of water with 5% acetonitrile and 0.1% formic acid. The mass spectrometric analysis of the samples was performed using an Orbitrap Velos Pro mass spectrometer (ThermoScientific). An ultimate nanoRSLC-HPLC system (Dionex) equipped with a custom end-fritted 50 cm × 75 µm C18 RP column filled with 2.4 µm beads (Dr. Maisch) was connected online to the mass spectrometer through a Proxeon nanospray source. 1–15 µl (depending on peptide concentration and sample complexity) of the tryptic digest was injected onto a 300 µm ID × 1 cm C18 PepMap pre-concentration column (Thermo Scientific). Automated trapping and desalting of the sample was performed at a flow rate of 6 µl/min using water/0.05% formic acid as solvent. Separation of the tryptic peptides was achieved with the following gradient of water/0.05% formic acid (solvent A) and 80% acetonitrile/0.045% formic acid (solvent B) at a flow rate of 300 nl/min: holding 4% B for 5 min, followed by a linear gradient to 45%B within 30 min and linear increase to 95% solvent B in additional 5 min. The column was connected to a stainless steel nanoemitter (Proxeon, Denmark), and the eluent was sprayed directly toward the heated capillary of the mass spectrometer using a potential of 2300 V. A survey scan with a resolution of 60,000 within the Orbitrap mass analyzer was combined with at least three data-dependent MS/MS scans with dynamic exclusion for 30 s either using CID with the linear ion-trap or using HCD combined with Orbitrap detection at a resolution of 7500. Data analysis was performed using Proteome Discoverer 2.2 (ThermoScientific) with SEQUEST search engine or MaxQuant with Andromeda search engine. Uniprot databases were used.

## Additional files

**Additional file 1.** List of histone peptides and ubiquitination sites identified by performing mass spectrometry analyses of ubiquitination reactions using SHPRH, UBE2D1 and recombinant nucleosomes.

**Additional file 2.** Mass spectrometry analyses of Ub chains (Ub-Ub linkages) from reactions using SHPRH, UBE2D1 and nucleosome substrates that were either assembled using HeLa or recombinant histones.

**Additional file 3.** Sites of SHPRH self-ubiquitination which were identified by mass spectrometry analyses of ubiquitination reactions using SHPRH and UBE2D1, in the presence of nucleosomes.

## Data Availability

The datasets used and/or analyzed during the current study are available from the corresponding author on reasonable request.
